# Patients expectation strongly associated with patients perception to nursing care: hospital based cross sectional study

**DOI:** 10.1186/s13104-018-3447-x

**Published:** 2018-05-18

**Authors:** Alem Girmay, Tekleweyni Marye, Mebrahtu Haftu, Dawit G/her, Tsion Brhanu, Hadgu Gerensea

**Affiliations:** School of Nursing, College of Health Science, Aksum University, Axum, Ethiopia

**Keywords:** Patient’s perception, Nursing care, Associated factors

## Abstract

**Objective:**

Nursing care is one of the most important components of health care and patient expectation toward nursing care is being rising. Accordingly, patients’ expectation needs to be managed adequately in order to improve outcomes and decrease liability through their perception. To improve the outcome based on the expectation of patients, we need to consider patients’ perception to the care they received. So this study aims to identifies the perceptions toward nursing care and their associated factors.

**Result:**

From a total of 281 admitted patients 151 (53.7%) were females; 136 (48.4%) were found in the age group of 21–30 years with mean age of 30 (11 ± SD) years. The mean score of overall perception were 62.6 ± 17.9 (95% CI 60.79–64.37). Among all 154 (54.8%) participants had poor perception to nursing care. Occupation, ward and expectation had association with perception. Patient’s level of perception towards nursing care was poor (54.7%) and ward where patients admitted, expectation of patients, occupation of patients and duration of hospital stay were significantly associated with patients’ perception. So that health institutions and nurses should focus on perception of their clients.

## Introduction

Fundamentally, patients express their requirements in terms of what they need, want, prefer and demand with respect to the nursing care they receive [1]. The requirements of patients could be considered as a demand for quality nursing care that nurses and health system attempt to meet [2–4]. Excellence in nursing care is what those in need of healthcare services wish for, and it is also the main goal for those providing the care [4–6].

To improve the quality of nursing care that is the combined product of patient expectation and perception, health care team especially the nurse should be aware what the patients perceive to nursing care service as well as what factors influence patients perception to nursing care [1].

Different studies revealed that nurses treated patients and relatives of hospitalized patients as inferior, nurses were rude and offered cold reception in public hospitals [7], and patients were dissatisfied with information provided because they felt the information given about their condition or related to their disease was inadequate [8].

A study done in Jordanin 2005 found out that majority of participants had positive experiences regarding time nurses spent with patients as well as respect for patients. It is not only the time nurses spent with patients and respect for patients that would determine patients perceptions of nursing care but also other factors such as adequate information about their condition and treatment, kindness, cheerfulness and other related things are expected from the nursing care [8, 9].

In another study conducted in Tanzania National Hospital revealed that patients were dissatisfied with the attitude of health professionals including nurses [7].

Current study reveals that while a very high percentage of patients have a good perception of the ward organization, capabilities, availability and responsiveness of the nurse quiet a fair percentage of patients perceive explanation and information and caring attitude qualities of the nursing care as poor. Many other studies have also revealed the patients perception of communication and information aspects of nursing care as less than adequate [10].

Still researchers said patients are satisfied or dissatisfied by using their own criteria which have no consistency. For this reason the health system is being blamed by patients at different time with different complaints including the whole community because there is a gap between the reports and actual patient expectation and perception on nursing care. Due to this, the image of nursing care was became ambiguous to patients parallel to this it also being disturbing the image of nursing professionals which influences the quality of the whole health system [11, 12].

Researches were done in different related contexts but few of them tried to dig out level of perception of patients with their associated factors to nursing care [3, 11]. Understanding patient’s perceptions towards nursing care enables health institutions to design nursing care based on the finding. It helps in identifying areas of patients’ satisfaction and dissatisfaction for the different items of nursing care. The result also may be used in quality assurance program of the institution, to health planners and to prepare training based on the findings and finally it will be a guide for further related studies. So this study assessed the perception of patients towards nursing care and its associated factors.

## Main text

### Study area and study design

Quantitative Hospital based cross sectional study was conducted from April 1 to 30, 2017 at Hiwot Fana Specialized University Hospital which is found in Harar Town, 525 km away from Addis Ababa. It provides a broad range of medical services to both in and out patient of all age groups with a total capacity of about 161 in patient beds in four major departments and other specialty units with average annual bed occupancy rate 72.2% and average annual length of stay 4.1 (5.4 for April) with annual turnover rate of 7 and 5 for April.

### Sample size

A total of 281 patients from medical, surgical, obstetrics and gynecology wards who was age greater or equal to 18 years old and admitted for at least 48 h were taken and interviewed randomly. Single population proportion for patients’ perception and associated factors were used to calculate the sample size, with assumptions; prevalence = 37.5% [13], CI at 95%, margin of error 4% for perception and power = 80%, the ratio of unexposed to exposed almost equivalent to 1 in Epi Info version 7 for the associated factors.

### Data collection tool and analysis

Pretested semi structured data collection tool which had five Likert Scales was used to collect data. To control quality of the data double data entry was done by two data clerks and consistency of the entered data were cross checked by comparing the two separately entered data on Epi Data. The raw data was cleaned, coded and entered into the computer as soon as data generated and analyzed using SPSS version 22. Data was summarized using descriptive and inferential statistics. Bivariate analysis at 95% confidence interval was used to infer association between the independent and outcome variables. Independent factors, with a p-value < 0.25 obtained in the bivariate logistic regression was entered into the multiple logistic regression model to obtain the significant variables. Statistical significance was declared at (p < 0.05). Variables which were statistically significant at p-value < 0.05 was identified as factors of patients perception to nursing care.

### Operational definitions

*Patient’s perception to nursing care*; the patient’s view on nursing care after nursing care received.

*Good perception*; having score above mean score of nursing care items.

*Poor perception*; having score below mean score of nursing care items.

### Ethics approval and consent to participate

Institutional Health Research Ethics Review Committee (IHRERC) of Haramaya University, College of Health and Medical Sciences, reviewed this study to ensure its protection. Following the approval by IHRERC of Haramaya University, official permission was also received from Hiwot Fana Specialized University Hospital (HFSUH). The objective and importance of the study was explained to the study participants. Data was collected after full informed oral consent was obtained from participants. In order to keep confidentiality of any information obtained, the data collection procedure was treated anonymous.

### Result

#### Sociodemographic characteristics and other relevant informations of participants

A total of 281 admitted patients were included in this study with a response rate of 100%. Out of these, 151 (53.7%) were females; 136 (48.4%) were found in the age group of 21–30 years with mean age of 30 (11 ± SD) years; 112 (39.9%) were from obstetrics and gynecology ward; 211 (75.1%) participants were married; 140 (49.8%) had not attended any formal education; 157 (55.9%) were from urban; 205 (73%) were Muslim in their religion and 84 (29.9%) were farmers in their occupation; 65 (23.1%) had history of previous hospitalization (Table 1).

**Table 1:** Sociodemographic characteristics and other relevant data of the study participants at Hiwot Fana Specialized University Hospital (HFSUH), Eastern Ethiopia, February 2017 (n = 281)

No	Sociodemographic characteristics and other information	Frequency	Percent (%)
1	Sex		
	Male	130	46.3
	Female	151	53.7
2	Religion		
	Orthodox	71	25.3
	Muslim	205	73
	Protestant	5	1.8
3	Residence		
	Rural	124	44.1
	Urban	157	55.9
4	Marital status		
	Single	54	19.2
	Married	211	75.1
	Divorced	13	4.6
	Widowed	3	1.1
5	Educational level		
	Noformaledu	140	49.8
	Grade 1–4	28	10
	Grade 5–8	53	18.9
	Grade 9–12	41	14.6
	College and above	19	6.8
6	Occupation		
	Civil servant	19	6.8
	Student	65	23.1
	Farmer	84	29.9
	Merchant	52	18.5
	Causal laborer	41	14.6
	Unemployed	20	7.1
7	Previous hospitalization		
	Yes	65	23.1
	No	216	76.9
8	Ward admitted		
	Medical	86	30.6
	Surgical	83	29.5
	Obs	37	13.2
	Gyn	75	26.7
9	Age (years)		
	< 20	61	21.7
	21–30	136	48.4
	31–40	41	14.6
	41–50	25	8.9
	> 50	18	6.4
10	Duration of stay (in days)		
	2–7	245	87.2
	8–14	30	10.7
	> 14	6	2.1

#### Patient’s perception towards nursing care

Patient’s perception towards nursing care were measured using 22 items which had five Likert Scale response that includes comprehensive nursing care components, physical nursing care components, psychological components and ethical performance and characteristics of nursing care components.

The Likert Scale result revealed that the mean rating values ranged from 2.06 (± 0.0809) to 3.5 (± 1.1). Responsiveness and cheerfulness of nursing care provision was rated the highest 3.5 (± 1.1) followed by sedating patients pain 3.28 ± 1.17, kindness and politeness of the service 3.23 ± 1.8

The mean score of overall perception were 62.6 ± 17.9 (95% CI 60.79–64.37). Those participants scored below the mean value taken as poor level of perception to nursing care and those participants scored above the mean score taken as good level of perception to nursing care. As to this 154 (54.8%) participants had poor perception to nursing care (Table 2).

**Table 2:** Level of patients perception towards nursing care in Likert Scale at Hiwot Fana Specialized University Hospital (HFSUH), Eastern Ethiopia, Feb 2017 (n = 281)

Items	Strongly disagree	Disagree	Neutral	Agree	Strongly agree	Total	Mean rating
Education about the disease and disease related issues to patients	34 (8.8)	238 (61.8)	40 (10.4)	55 (14.3)	18 (4.7)	940	2.44 ± .996
Responding to patients questions and giving information about the disease and medications	30 (7.8)	116 (30.1)	127 (33)	89 (23.1)	23 (6)	1114	2.89 ± 1.37
Meeting the patients needs	28 (7.3)	71 (18.4)	125 (32.5)	121 (31.4)	40 (10.4)	1229	3.19 ± 1.082
Sedating the patients pain	35 (9.1)	47 (12.2)	94 (24.4)	136 (35.3)	73 (19)	1320	3.43 ± 1.191
Following up the patients	47 (12.2)	65 (16.9)	94 (24.4)	119 (30.9)	60 (15.6)	1235	3.21 ± 1.245
Frequent presence around the patients and asking how they feel	50 (13)	87 (22.6)	95 (24.7)	102 (26.5)	51 (23.2)	1172	3.04 ± 1.242
Immediate response to their needs	66 (17.1)	117 (30.4)	93 (24.2)	70 (18.2)	39 (10.1)	1054	2.74 ± 1.23
Paying attention to the patients’ individual needs	85 (22.1)	135 (35.1)	89 (23.1)	53 (13.8)	23 (6)	949	2.46 ± 1.152
Providing comfort patients	92 (23.9)	129 (33.5)	67 (17.4)	61 (15.8)	36 (9.4)	975	2.53 ± 1.268
Keeping patient privacy and screening during procedure	123 (31.9)	113 (29.4)	58 (15.1)	56 (14.5)	35 (9.1)	922	2.39 ± 1.311
Convincing the patients	29 (7.5)	76 (19.7)	123 (31.9)	112 (29.1)	45 (11.7)	1223	3.18 ± 1.109
Calmly speaking with them	43 (11.2)	64 (16.6)	129 (33.5)	105 (27.3)	44 (11.4)	1198	3.11 ± 1.155
Reassuring them	47 (12.2)	95 (24.7)	108 (28.1)	101 (26.2)	34 (8.8)	1135	2.95 ± 1.163
Not causing them stress	55 (14.3)	96 (24.9)	130 (33.8)	70 (18.2)	34 (8.8)	1087	2.82 ± 1.152
Gaining their trust	76 (19.7)	120 (31.2)	87 (22.6)	63 (16.4)	39 (10.1)	1024	2.66 ± 1.248
Creating relationships with them	107 (27.8)	90 (23.4)	81 (21)	64 (16.6)	43 (11.2)	1001	2.6 ± 1.343
Listening to them	107 (27.8)	114 (29.6)	57 (14.8)	63 (16.4)	44 (11.4)	978	2.54 ± 1.35
Thinking for the patients	148 (38.4)	90 (23.4)	63 (16.4)	47 (12.2)	37 (9.6)	890	2.31 ± 1.345
To be kind, polite, friendly and respectful	25 (6.5)	90 (23.4)	81 (21)	130 (33.8)	59 (15.3)	1263	3.28 ± 1.17
Harsh and rude	73 (19)	247 (64.2)	40 (10.4)	17 (4.4)	8 (2.1)	795	2.06 ± .809
Knowledgeable and competent	34 (8.8)	51 (13.2)	140 (36.4)	111 (28.8)	49 (12.7)	1245	3.23 ± 1.11
Responsive and cheerful	30 (7.8)	34 (8.8)	96 (24.9)	165 (42.9)	60 (15.6)	1346	3.5 ± 1.1
Likert Scale mean score of perception							2.78 ± 0.95
Overall mean score of perception						24,095	62.6 ± 17.9
Overall how do you rate nursing care	Excellent	63 (16.4%)				925	2.4 ± .928
	Good	160 (41.6%)					
	Fair	106 (27.5%)					
	Poor	56 (14.5%)					

Frequency and percent of participant response [number in bracket indicates percent (%)]

#### Factors associated with patient’s perception towards nursing care

Variables such as age of participants, sex, educational level of participants, marital status, religion, previous hospitalization history, ward and patients expectation were inserted to the binary logistic regression to see variables those have association with the outcome variable, then after wards where patients admitted, occupation, sex, duration of hospital stay and expectation of patients had association with patients perception towards nursing care with p-value ≤ 0.25 in the binary logistic regression. To control multicolinearityVIF was used. Wards where patients admitted, occupation, duration of hospital stay and expectation of patients had significant association with the patients level of perception towards nursing care in the multivariate logistic regression model with p-value less than 0.05 (Table 3).

**Table 3:** Factors associated towards patients perception to nursing care at Hiwot Fana Specialized University Hospital (HFSUH), Eastern Ethiopia, Feb 2017 (n = 281)

Independent variables	Frequency	Level of perception		p-value	COR 95% CI	p-value	AOR 95% CI
		Good	Poor				
Sex							
Female	151	72	79	0.211	0.77 (0.513–1.158)	0.542	0.844 (0.489–1.456)
Male	130	55	75	1		1	
Occupation							
Civil servant	19	9	10	0.269	1.853 (0.62–5.526)	0.373	1.685 (0.535–5.312)
Student**	65	26	39	0.175	1.547 (0.60–3.974)	0.032**	1.196 (1.132–3.312)
Farmer	84	44	40	0.130	2.011 (0.81–4.96)	0.237	1.788 (0.68–4.685)
Merchant	52	22	30	0.234	1.788 (0.68–4.657)	0.368	1.586 (0.58–4.328)
Causal labor	41	19	22	0.196	1.929 (0.713–5.21)	0.358	1.630 (0.57–4.62)
Unemployed	20	7	13	1			
Ward admitted							
Medical**	86	31	55	0.001	0.357 (0.20–0.62)	0.009**	0.416 (0.21–0.80)
Surgical	83	41	42	0.155	0.074 (0.39–1.16)	0.364	0.729 (0.37–1.44)
Gynecology**	37	23	14	0.003	0.352 (0.17–0.702)	0.001**	0.268 (0.12–0.58)
Obstetrics	75	41	34	1			
Patient expectation							
Good**	172	74	98	0.019	2.5 (1.146–5.378)	0.01**	13.146 (11.31–17.5)
Poor	109	53	56	1			
Duration of stay in hospital (days)							
2–7	245	108	137	0.011	3.3 (1.89–6.43)	0.001**	1.21 (0.98–1.56)
8–14	30	16	14	0.023	1.5 (1.25–2.24)	0.02**	3.4 (1.74–5.97)
> 14	6	3	3	1			

** Variables having significance difference to patients perception towards nursing care

### Discussion

#### Patient’s perception towards nursing care

Patients had also reflected their perception in five Likert Scale on the nursing care, as to this responsiveness and cheerfulness of nursing care provision was rated the highest 3.5 (± 1.1). Sedating patients pain, kindness and politeness of the service, knowledgeable and competence of nursing care, patient follow up scored highest next to responsiveness and cheerfulness (3.43 ± 1.19, 3.28 ± 1.17, 3.23 ± 1.11, 3.21 ± 1.245 respectively). Harsh and rude, thinking for patients, privacy provision, education on disease and disease related conditions, paying attention to patients individual needs got lowest score as compared from the above (2.06 ± 0.809, 2.31 ± 1.345, 2.39 ± 1.31, 2.44 ± 0.996, 2.46 ± 1.152 respectively). This finding was different from the finding in Turkey in 2009 and Iran in 2005 [14, 15], in this study there were high score other than physical care. This may due to the difference in study period, study area and living standard.

The mean score of perception to nursing care in Likert Scale was 2.78 ± 0.95, this was lower than from the finding of study done in China in 2011 [16], which was 4.14 ± 0.62. The mean score of overall total perception to nursing care components were 62.6 ± 17.9, this finding were lower than the finding of study done in Iran in 2015 [17] and study done in Nepal in 2014 [18] which were 93.33 ± 12.43 and 97.32 ± 13.45 respectively, this may be happen due to study area difference.

56.1% of participants had poor perception to nursing care. This finding were different from the study done in Ethiopia, Mekelle in 2013 [13] which was 9.4% poor perception. This may be due to the instrument used to measure level of perception and study area.

#### Factors associated with patient’s perception towards nursing care

Wards where patients admitted had significant association with patient’s perception towards nursing care. Being treated in medical ward had 0.416 times less perception than other wards (AOR = 0.416; 0.215, 0.806). This finding was different from the previous findings in studies done in Britain in 2001 [19], Brazil in 2009 [15] and Iran in 2013 [11]. This may be due to difference in study period, study area and hospital wards infrastructure.

Patient’s expectation had significant association towards nursing care. Patients having good expectation towards nursing care had 13 time more perception than patients having poor expectation (AOR = 13.146; 11.313, 17.538). This finding was similar with the study done in Philippine 2013 [1].

Occupation had significant association with patient’s perception towards nursing care. Being student was 1.2 times more perception than other occupations (AOR = 1.196; 1.132, 3.312). This finding was similar with study done in Iran in 2013 [11].

Educational status, previous hospitalization, residence and age had positive association with patient’s perception towards nursing care even though not significant. This was similar with study in Britain in 2001 [19].

### Conclusion

Patient’s level of perception towards nursing care was poor (54.7%) and ward where patients admitted, expectation of patients and occupation of patients were factors significantly associated with patients’ perception. So that health institutions and nurses should focus on perception of their clients.

### Limitation of the study

Since the study was conducted in specific area the cultural and social norms may affect the result so that it should be repeated in different population. Moreover, the study design may not show cause and effect so that it should be repeated with different study design.

## Abbreviations

CI: confidence interval
AOR: adjusted odd ratio
SPSS: Statistical Package for Social Sciences
